# A review on disinfection methods for inactivation of waterborne viruses

**DOI:** 10.3389/fmicb.2022.991856

**Published:** 2022-09-23

**Authors:** Adedayo Ayodeji Lanrewaju, Abimbola Motunrayo Enitan-Folami, Saheed Sabiu, Feroz Mahomed Swalaha

**Affiliations:** Department of Biotechnology and Food Science, Durban University of Technology, Durban, South Africa

**Keywords:** chlorination, disinfection, disinfection by-products, viral inactivation, waterborne viruses

## Abstract

Water contamination is a global health problem, and the need for safe water is ever-growing due to the public health implications of unsafe water. Contaminated water could contain pathogenic bacteria, protozoa, and viruses that are implicated in several debilitating human diseases. The prevalence and survival of waterborne viruses differ from bacteria and other waterborne microorganisms. In addition, viruses are responsible for more severe waterborne diseases such as gastroenteritis, myocarditis, and encephalitis among others, hence the need for dedicated attention to viral inactivation. Disinfection is vital to water treatment because it removes pathogens, including viruses. The commonly used methods and techniques of disinfection for viral inactivation in water comprise physical disinfection such as membrane filtration, ultraviolet (UV) irradiation, and conventional chemical processes such as chlorine, monochloramine, chlorine dioxide, and ozone among others. However, the production of disinfection by-products (DBPs) that accompanies chemical methods of disinfection is an issue of great concern due to the increase in the risks of harm to humans, for example, the development of cancer of the bladder and adverse reproductive outcomes. Therefore, this review examines the conventional disinfection approaches alongside emerging disinfection technologies, such as photocatalytic disinfection, cavitation, and electrochemical disinfection. Moreover, the merits, limitations, and log reduction values (LRVs) of the different disinfection methods discussed were compared concerning virus removal efficiency. Future research needs to merge single disinfection techniques into one to achieve improved viral disinfection, and the development of medicinal plant-based materials as disinfectants due to their antimicrobial and safety benefits to avoid toxicity is also highlighted.

## Introduction

Worldwide, there is an urgent demand for potable water as approximately 1 in 4 people of the global population have no access to safe drinking water in the year 2020 (WHO, UNICEF, 2021). At the beginning of the COVID-19 pandemic, three out of 10 individuals in the world could not wash their hands with soap and clean water in their homes. Unfortunately, it has been estimated that by the year 2030, about 19% of the global population (1.6 billion persons) will not have access to safe water (WHO, UNICEF, 2021).

Waterborne diarrhea is a prominent global morbidity and mortality source, with an estimated 4 billion instances of sickness and 1.8 million deaths annually (Manetu and Karanja, 2021). Approximately 90% of death associated with diarrhea globally resulted from poor hygiene, inadequate sanitation, and, more importantly consumption of unsafe water. Apart from the loss of lives linked with poor access to safe water, it also leads to a global economic loss of about US$260 billion *per annum* (WHO, 2012). Unsafe water could contain bacteria, protozoa, and viruses which are implicated in several human diseases with gastroenteritis being the most notable among them (Gall et al., 2015). Unfortunately, waterborne viruses, which are more persistent in the environment than bacteria, are frequently the source of diarrheal sickness in drinking, recreational, and groundwaters (Magana-Arachchi and Wanigatunge, 2020). However, less attention is given to these viruses despite their huge negative impact on public health (Gall et al., 2015; Lanrewaju et al., 2022).

Most viruses are linked to gastroenteritis resulting in diarrhea and other symptoms such as abdominal cramping, vomiting, and fever (Magana-Arachchi and Wanigatunge, 2020). Furthermore, they could be responsible for more severe health conditions such as encephalitis, myocarditis, meningitis, cancer, and hepatitis among others (WHO, 2016; Magana-Arachchi and Wanigatunge, 2020; Lanrewaju et al., 2022). Unfortunately, few antiviral drugs with a broad spectrum of action to treat these diseases are available. Both symptomatic and asymptomatic patients can excrete a significant quantity of viruses. Clinical observations have shown that infected individuals with symptomatic infection may shed viruses for a few weeks after infection (Albinana-Gimenez et al., 2006; Wu et al., 2020; Yeo et al., 2020).

Primarily, waterborne enteric viruses are transmitted through the fecal-oral route (Sánchez and Bosch, 2016; Bouseettine et al., 2020) which could be from person to person or *via* drinking contaminated water with its accompanying health hazard (Diaz, 2006; Upfold et al., 2021). Discharge of influents generated from faeces, vomit, and urine of infected animals and humans could introduce viruses into wastewater sources (Bosch et al., 2006; Diaz, 2006). Viruses that could be detected in wastewater include adenoviruses (AdVs), enterovirus (EVs), polioviruses (PVs), hepatitis A viruses (HAV), hepatitis E viruses (HEV), rotaviruses (RVs), reoviruses, noroviruses (NoVs), and coronaviruses (including SARS-CoV-2) among others (Diaz, 2006; Kitajima et al., 2014; Farkas et al., 2018; Gormley et al., 2020; Buonerba et al., 2021; Hasan et al., 2021; Lanrewaju et al., 2022). Virus genome copies (GC) L^−1^ approximately range from 10^5^ to 10^7^ in raw domestic wastewater (Albinana-Gimenez et al., 2006). The ongoing global outbreak of SARS-CoV-2 which is responsible for COVID-19 has raised the urgency of elucidating the fate and prevalence of coronaviruses as well as other viruses in sewage and drinking water sources.

Viruses can be reduced by 3-4log with traditional primary and secondary wastewater treatment (Sidhu et al., 2018); however, several viruses may survive in treated effluent and then contaminate natural water sources when discharged into it. The minimum requirement for the water treatment method to be considered efficient for viral disinfection is to achieve a 99.99% (4log) reduction of viral concentrations in water after treatment as recommended by the United States Environmental Protection Agency and Health Canada (Monteiro et al., 2015). Hence, viral contamination is a major concern when the effluent is discharged into freshwater sources, or when insufficient viral elimination occurs during planned or accidental water recycling (Chen et al., 2021b). One of the challenges of the traditional water/wastewater treatment processes is the low removal efficiency due to virus sizes; however, adopting membrane-based technologies helps overcome this challenge (Ibrahim et al., 2021). Furthermore, free chlorine, a commonly used disinfectant, has been linked with the formation of regulated toxic disinfection by-products (DBPs). This has led to the adoption of other disinfectants such as monochloramine, chlorine dioxide, and ozone among others by some drinking water utilities. Recently, there has been a search for holistic disinfection techniques with much lower health risks. Unfortunately, this ongoing search comes with costs and operational challenges (Gall et al., 2015). Therefore, the objective of this study is to critically review different disinfection methods, and highlight some of their merits and limitations associated with the inactivation of waterborne viruses.

## Disinfection methods for the inactivation of waterborne viruses

**Figure 1:**
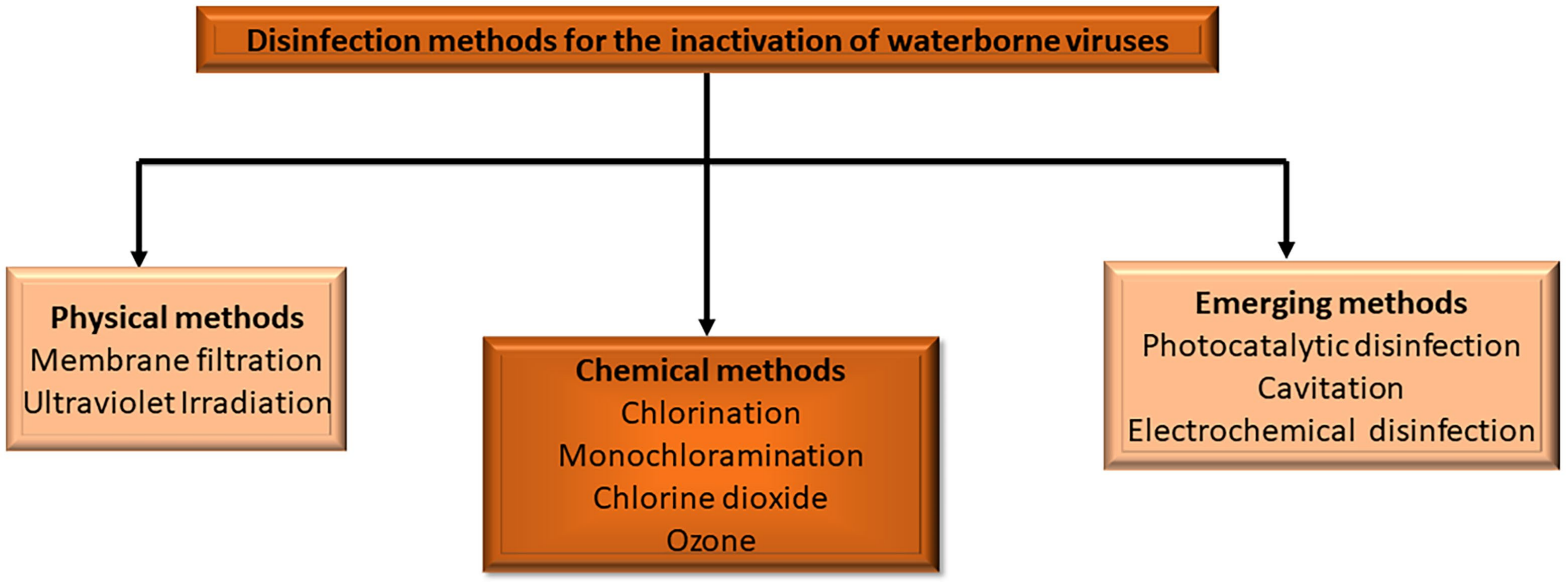
Disinfection methods for the inactivation of waterborne viruses (adapted from Chen et al., 2021b).

Effective disinfection strategies are vital in water treatment procedures because they ensure the elimination of pathogenic microorganisms responsible for waterborne illnesses. The commonly used techniques of disinfection for viral inactivation in water comprise conventional chemical processes such as chlorine, monochloramine, chlorine dioxide, and ozone (Figure 1). Furthermore, membrane filtration and UV irradiation which are physical methods have also been utilized for disinfection in the treatment of water (Collivignarelli et al., 2018). Many parameters, including water, pH, temperature, type of microorganisms, type of disinfection, disinfectant dose, contact time, and inorganic and organic material in water, are known to influence disinfection (Tsitsifli and Kanakoudis, 2018). Even though disinfection entails the process of pathogen inactivation, the use of chemical disinfectants can result in the production of inorganic and organic DBPs (Sadiq and Rodriguez, 2004). Interestingly, the introduction of improved disinfection technologies such as advanced oxidation processes (AOPs) is a viable strategy for enhancing the water and wastewater treatment processes (Feitz, 2005; Kokkinos et al., 2021).

Advanced oxidation processes have been considered a potential, environmentally acceptable, and effective alternative to the traditional disinfection approaches for controlling the microbiological quality of water. Water disinfection and degradation of different toxic pollutants are achieved through the on-site production of chemical oxidants (Shabat-Hadas et al., 2017; Giannakis et al., 2017b; Marjanovic et al., 2018; Rekhate and Srivastava, 2020). Generally, AOPs are redox technologies that involve various oxidation processes, including ozonation, ozonation coupled with hydrogen peroxide (H_2_O_2_) and/or UV radiation, photocatalysis activated by semiconductors such as TiO_2_, and electrochemical oxidation among others (Figure 1). Their mechanism of action is a function of the formation of very reactive oxygen species (ROS) that are not target-specific and can be utilized as a pre-or post-treatment to a biological procedure (Galeano et al., 2017; Kokkinos et al., 2020). Therefore, photocatalytic, cavitation, and electrochemical methods of disinfection are highlighted as emerging methods of disinfection in this review.

Meanwhile, the safety of treatment plant operators should be considered whenever disinfectants are used because some of the disinfectants are harmful; hence, compliance with safety precautions by the operators handling the disinfectants is important. For instance, UV radiation is considered a “complete carcinogen” because it is both a mutagen and a nonspecific damaging agent. In addition, it can initiate and promotes tumor formation (D’Orazio et al., 2013; Raeiszadeh and Adeli, 2020). Likewise, ozone can harm the lungs when inhaled. In relatively little doses, chest pain, coughing, shortness of breath, and throat irritation can occur. Additionally, ozone may weaken the body’s defenses against respiratory infections and aggravate chronic respiratory conditions like asthma (USEPA, 2008). Therefore, appropriate personal protective equipment (PPE), such as goggles, gloves, long-sleeved shirts, long pants, and masks should be worn while applying disinfectants (Dhama et al., 2021). Using a suitable mask for a given purpose is preferable to using any non-specific mask (Agrawal et al., 2020).

## Physical methods

### Membrane filtration

Membrane filtration is a highly efficient technique for the removal of suspended particles, bacteria, and organic materials from drinking water and wastewater. This method enables the separation of contaminants present in water by passing it through a physical barrier. Commonly used technologies are microfiltration (MF), ultrafiltration (UF), nanofiltration (NF), and reverse osmosis (RO; Chen et al., 2021a). The basic mechanism of virus removal by membrane filtration is size exclusion, as ultrafiltration with a nominal pore size of 10^−2^ μm is suitable for the removal of most viruses (Zhang et al., 2016). Apart from the principle of size exclusion which operates on the surface of the membrane, other mechanisms involving electrostatic interactions are linked with the virus and membrane charge, adsorption retention, and hydrophilic-hydrophobic reactions. They are also associated with the physicochemical parameters of the membranes as well as the viruses. These properties facilitate the movement of the viruses through the surface of the membrane and their deposition on the internal matrix of the membrane (Van Voorthuizen et al., 2001; Schaldach et al., 2006; ElHadidy et al., 2013; Gentile et al., 2018; Chen et al., 2021a). However, the effectiveness of the membrane is affected by the quality of water, the content of solid particles in the water, and fouling formation during the water treatment process (Collivignarelli et al., 2018). Therefore, several membrane modifications have been made to enhance its efficiency, and consequently, this has resulted in the inactivation of viruses as the modifications facilitate the incorporation of materials with enhanced antiviral properties (Sinclair et al., 2018).

Membranes have been modified with different materials such as polymers and in the field of antimicrobial polymers, polyethylenimine (PEI) is one of the most investigated amine-containing polymers (Jarach et al., 2020). Sinclair et al. (2018) modified a commercial polyether sulfone (PES) microfiltration membrane using cationic polyethylenimine (PEI) for virus removal through gravity filtration. The virus removal rate was enhanced as the membrane modification weakened the virus build-up on the membrane surface *via* surface repulsion. In addition, the utilization of the modified membrane required additional force at a reduced cost yet increased the rate of virus removal. In another study, cross-linked multilayers on model surfaces and commercial PES MF membranes were created using a cross-linking agent; terephthalaldehyde (TA), for the formation of a positively charged membrane for trapping and inactivation of viruses. Furthermore, silver and copper nanoparticles (Ag and CuNPs) with antiviral activity were coated on the substrates and stabilized with PEI. These resulted in a 4.5-5log unit reduction of MS2 bacteriophages through the viral particle inactivation and adsorption (Sinclair et al., 2019). Therefore, the incorporation of nanoparticles has enhanced the virus removal efficiency as well as the reinforcement of the structural integrity, membrane hydrophilicity, and electrostatic interaction between the viruses and the membrane for increased water flux (Khin et al., 2012; Liu et al., 2019; Németh et al., 2019; Kuo et al., 2020; Chen et al., 2021a).

Two major challenges in water treatment using membrane filtration are biofouling and virus penetration, as the membrane’s permeability decreases with increased biofouling. Consequently, it results in high energy costs and a reduction in the membrane’s durability (Zodrow et al., 2009). Furthermore, virus separation is increased to some extent by membrane fouling (Chen et al., 2021a); however, the membrane surface could become weakened accompanied by increased penetrability and then compromised virus retention (Wang et al., 2017). Membranes have been modified with different materials such as polymers; however, polymer leaching is a problem associated with this technology (Sinclair et al., 2018) which could lead to a loss of the antiviral ability of the material when the substance is leached into the environment (Koplin et al., 2008; Sinclair et al., 2019). Another material that has been incorporated into the membrane for its modification is nanoparticles due to their proven antimicrobial properties; however, the loss of nanoparticles is a major drawback (Zodrow et al., 2009). Therefore, further research is needed in this regard to concentrate on the encapsulation of the nanoparticles in a bid to control their release.

### Ultraviolet irradiation

One of the physical techniques used for the inactivation of microorganisms is ultraviolet radiation (Oguma and Rattanakul, 2021). The International Commission on Illumination has described the UV region of the electromagnetic spectrum as radiation with wavelengths that ranges from 100 to 400 nm (CIE, 2003; ASHRAE, 2019). Furthermore, the UV spectrum is categorized into UVA (wavelengths of 400–315 nm), UVB (315–280 nm), UVC (280–200 nm), and vacuum UV (VUV; 200–100 nm; IESNA, 2000). A relatively high energy level is associated with radiation in the UV range, and microbes absorb protons with a high absorption coefficient between 200 and 300 nm (Chen et al., 2021a). Exposure of microorganisms to UV light damages their nucleic acids (DNA or RNA; Ibrahim et al., 2021). In other words, the process entails running water through UV disinfection tubes, which damages the nucleic acids resulting in the non-viability of the bacteria and viruses, thus, become incapable of reproduction (Wigginton et al., 2012; Sigstam et al., 2013). This disinfection method has been proven effective for the inactivation of cysts of *Cryptosporidium* and *Giardia* in water (Chen et al., 2021b). The UV disinfection is considered an efficient and competitive alternative for the disinfection of secondary effluent because it does not make use of chemical agents, is non-corrosive, easy to install and operate, and produces no DBPs, hence no toxic residue after disinfection (Tondera et al., 2015; Zhang et al., 2016). Short contact time is required for disinfection due to the rapid-reacting nature of the radicals, and there is no odor or taste when it is used. Therefore, UV radiation is appropriate for drinking water treatment plants (Ibrahim et al., 2021).

The most frequently used UV sources are low-pressure (LP) and medium-pressure (MP) UV. They are mercury lamps for monochromatic UV at 254 nm and polychromatic UV with a broad spectrum, respectively, (Song et al., 2019). Germicidal lamps produce UVC energy by emitting radiation mostly at a wavelength of 253.7 nm, which kills or inactivates microorganisms (ASHRAE, 2019). Viruses are inactivated differently due to the morphological and genomic varieties (enveloped or non-enveloped; DNA or RNA viruses) of the different viral species as well as the wavelength of the germicidal UV applied (Oguma and Rattanakul, 2021). For instance, DNA replication in viruses is generally inhibited by UV at 254 nm and 280 nm irradiation. However, UV at 224 nm irradiation has little effect on the viability of the human adenovirus-2 (HAdV-2) genome. Nevertheless, the impact of capsid alterations may impede the virus’s genome ability to enter the nucleus in host cells (Vazquez-Bravo et al., 2018). Conversely, 3log inactivation can be attained for poliovirus, coxsackievirus, and echovirus using UV at 254 nm in the range of 14 to 27 mJ/cm^2^ (Gerba et al., 2002; Shin et al., 2005).

In a bid to enhance viral inactivation using UV, H_2_O_2_, ozone, sodium hypochlorite, and chlorine dioxide, among others, have been incorporated into the UV disinfection process. Mamane et al. (2007), incorporated 25 mgl^−1^ dose of H_2_O_2_ to filtered UV irradiation for 15 min, which led to a 2.5log increase inactivation effect on MS2, whereas MS2 was resistant to only UV irradiation at >295 nm. The authors opined that hydroxyl radicals produced from the photolysis of H_2_O_2_ could be responsible for the observed effect and could have destroyed the cell membranes of MS2 with a simple but rigid structure. Similarly, combining UV treatment with chlorine resulted in 3-5log_10_ reductions for chlorine-resistant coliphages coupled with reduced DBPs formation (Zyara et al., 2016).

Ultraviolet C (UVC) light with wavelength between 207 and 222 nm has been reported to be effective for the inactivation of microorganisms. Specifically, far UVC light is considered safe for human health compared to the traditional germicidal UV light because it does not damage mammalian cells (Buonanno et al., 2017; Ponnaiya et al., 2018; Buonanno et al., 2020; Narita et al., 2020). Sequential UVA and UVC irradiation using light-emitting diodes (UV-LED) were applied for *Escherichia coli* and MS2 inactivation in a study carried out by Song et al. (2019). There was no improvement in the inactivation of MS2 as compared to *E. coli* following UVA pre-treatment accompanied by UVC treatment. The observed inactivation of MS2 in their study was attributed to its composition as being void of a cellular metabolic system (Song et al., 2019).

The efficiency of 222 nm UVC light for the inactivation of alpha HCoV-229E and beta HCoV-OC43 was evaluated using an aerosol irradiation chamber. Aerosolized coronavirus 229E and OC43 were inactivated by UVC with low doses of 1.7 and 1.2 mJ/cm^2^, respectively, (Buonanno et al., 2020). Biasin et al. (2021), investigated the ability of UVC irradiation at different doses to inactivate SARS-CoV-2 (Virus Human 2019-nCoV strain 2019-nCoV/Italy-INMI1, Rome, Italy) at a multiplicity of infection (MOI) of 1000, 5, and 0.05. The authors reported that UVC dose of just 3.7 mJ/cm^2^ was adequate to attain above 3log inactivation without viral replication. All the viral concentrations evaluated at a UVC dose of 16.9 mJ/cm^2^ were inactivated completely. Mariita et al. (2022), inactivated Feline calicivirus (FCV) ATCC VR-782; a surrogate of norovirus with a UVC light-emitting diode (LED) array (KL265-50 V-SM-WD). A 99.9% virus reduction (3log reduction) was achieved at a UVC dose of 22.5 mJ cm^−2^; hence, the authors posited that NoVs can be inactivated effectively using UVC LED array (Mariita et al., 2022).

Disinfection methods, especially UV light, have repeatedly been found ineffective against adenovirus (Prado et al., 2019); hence, optimizing this method for the inactivation of adenovirus is a necessity. The use of UV-LEDs for viral inactivation just got into the limelight recently (Rattanakul and Oguma, 2018; Keshavarzfathy et al., 2021); therefore, more research is needed in this regard. Although UV disinfection is known for not resulting in the formation of DBPs compared to chlorine, turbid and colored particles in the secondary effluent may reduce UV light penetration which affects its disinfection ability (Zhang et al., 2016). In addition, incorporation of H_2_O_2_ into UV irradiation for improved viral inactivation is less efficient when applied to wastewater with increased absorbance and its utilization is affected by the high cost of operation (Rasalingam et al., 2014).

## Chemical methods

### Chlorination

For several decades, chlorination has been used in both developing and advanced countries as the most economical, traditional, and versatile disinfectant for water disinfection processes (Lim et al., 2010; Hu et al., 2019; Martino, 2019; Luo et al., 2020; Rachmadi et al., 2020). The slower decay rate of chlorine in comparison to many other chemical disinfectants is responsible for its capacity to retain disinfection activity for longer periods; hence, its preference for use in drinking water distribution systems and water reservoirs avoids subsequent replication of the microorganisms (WHO, 1996; Xiao et al., 2020).

Chlorine interacts with organic chemicals in the water to generate several secondary products that are more hazardous than the parental components (Anand et al., 2014). Chlorine gas combines with water to generate aqueous HOCl and HCl (Equation 1), and then HOCl dissociates further to form OCl^−^ and H^+^ (Equation 2; Lin and Lee, 2007; Collivignarelli et al., 2018). The use of aqueous chlorine for disinfection is a function of contact time and concentration (Kumar et al., 2020).


(1) $${\mathrm{Cl}}_{2}+H_{2}O↔\mathrm{HOCl}+\mathrm{HCl}$$

(2) $$\mathrm{HOCl}↔{\mathrm{OCl}}^{-}+H^{+}$$

Chlorine gas and water have a reversible and pH-dependent chemical interaction in nature (Hung et al., 2017). The presence of OCl^−^ and HOCl in large quantities in water aids in the oxidation, hydrolysis, and disintegration of cell membranes. These reactions between the chlorine compounds and membrane proteins of the microorganisms in water result in the formation of chloro-nitrogen compounds. Proteins, DNA, lipids, and cholesterol are all biological targets that HOCl is known to interact within the body (Hawkins et al., 2003). The purine and pyrimidine sequences of the DNA of the microorganisms are altered by HOCl as this is expressed in its negative effect on the genetic sequence mutation, metabolism, synthesis of protein, and transport of glucose (Kumar et al., 2020).

Chlorine exhibits its potent bactericidal effect by blocking metabolic activities through a variety of complex processes. The modus operandi it adopts alters the chemical composition of the enzymes at the center of bacteria’s nutrition systems, rendering them inactive thereby preventing their growth and survival (Collivignarelli et al., 2018). However, some endospore-forming bacteria such as *Bacillus* and *Clostridium* are not inactivated by chlorine disinfection. At the same time, protozoa, including *Entamoeba histolytica* and *Giardia lamblia*, require a high dose (mgL^−1^) and considerable contact time to be inactivated (Kumar et al., 2020).

The destruction of viral capsid protein and the inhibition of genome replication can both be achieved toward the inactivation of viruses using free chlorine (Fuzawa et al., 2019). At a pH of 7.4, room temperature, 5 mM PO_4_^2−^, and 10 mM NaCl, it was reported that no undamaged capsid protein was detected after 5log inactivation of MS2 with chlorination having caused significant damage to the genome and capsid protein. The considerable capsid protein degradation led to a substantial loss of protein-mediated binding or injection capabilities, and the treatments hindered replication functions and causes significant genome damage (Wigginton et al., 2012).

The efficiency of free chlorine in the inactivation of waterborne viruses has been expressed to be temperature and pH-dependent (Lim et al., 2010) as well as the type of virus involved. Table 1 shows the estimated Ct values for 4log reduction of waterborne viruses using the efficiency factor hom (EFH) model. This method has been utilized to evaluate disinfection kinetics in drinking water to ensure conformity with the Surface Water Treatment Rules for microbiological reduction requirements (Haas and Joffe, 1994). The idea of disinfectant dose and contact time is essential to comprehending disinfection kinetics and using the Ct concept (Collivignarelli et al., 2018). The Ct values are the product of the disinfectant dose C (in mgL^−1^) and the contact time *t* (in minutes; Haas and Joffe, 1994; Girones et al., 2014; Rachmadi et al., 2020). They are an important metric for practical disinfection evaluation and system design (Collivignarelli et al., 2018; Chen et al., 2021b). Under similar conditions, Echovirus (E), Coxsackievirus B (CVB), and PV are more resistant to inactivation using chlorination. However, pH dependence for disinfection of CVB5, CVB6, PV, E-1, and E-12 was revealed to occur at pH 9 or higher, resulting in a remarkable increase in the required Ct values for the inactivation of these viruses when compared to pH 7 or lower without a change in the time (Black et al., 2009; Cromeans et al., 2010). In addition, it has been documented that reoviruses are more sensitive to chlorine compared to enteroviruses (Betancourt and Gerba, 2016). It has not been reported that chlorine effectively controls all waterborne viruses as Norovirus suspension remained infectious after 30 min of contact with 3.75 mgl^−1^ free chlorine (Keswick et al., 1985).

**Table 1:** Estimated Ct values for 4-logs reduction of waterborne viruses using the efficiency factor hom (EFH) model (adapted from Chen et al., 2021b).

Enteric virus	CT value (mg min L^−1^)	Disinfectant dose (mg L^−1^)	Conditions	References
Adenovirus-40	0.22	1.0	pH 6, 5°C	Thurston-Enriquez et al. (2003)
	0.75		pH 7, 5°C	
	0.27		pH 8, 5°C	
Poliovirus-1	6.36		pH 6, 5°C	
	5.3		pH 7.5, 5°C	
	5.3	1.0	pH 7.5, 5°C	Black et al. (2009)
	22.9	1.0	pH 9, 5°C	
Coxsackievirus B5	11.5	1.0	pH 7.5, 5°C	
	22.9	1.0	pH 9, 5°C	
Echovirus 1	6.2	1.0	pH 7.5, 5°C	
	16.6	1.0	pH 9, 5°C	
Echovirus 12	7.4	1.0	pH 7.5, 5°C	
	32.3	1.0	pH 9, 5°C	
Coxsackievirus B3	2.9	0.2	pH 7, 5°C	Cromeans et al. (2010)
	1.7	0.2	pH 8, 5°C	
Coxsackievirus B5	7.4	0.2	pH 7, 5°C	
	10	0.2	pH 8, 5°C	
MS2	0.435	0.172	pH 7.2, 5°C	Lim et al. (2010)
	0.183	0.172	pH 7.2, 20°C	
Rotavirus	5.55	0.4	pH 7.2, 20°C	Xue et al. (2013)
Adenovirus-2	1.65	2.7	pH 8, 25–26°C	Girones et al. (2014)

In the presence of naturally occurring organic molecules, chlorine produces trihalomethane and acetoacetic, which are recognized to be carcinogenic to humans with the most frequent being the trihalomethanes (THMs; Rebhun et al., 1997; Guay et al., 2005; Sun et al., 2009; Badawy et al., 2012; Wu et al., 2013; Sorlini et al., 2015; Sorlini et al., 2016). While chloroform, bromodichloromethane (BDCM), chlorodibromomethane (CDBM), and bromoform comprise the volatile compound category. The THMs, haloacetic acids (HAAs), chlorophenols, chloral hydrate, and haloacetonitriles (HANs) are some of the undesired halogenated organic compounds formed when chlorine combines with natural organic molecules (humic and fulvic acids). Although brominated THMs can be produced in large quantities when waters with high bromide content are chlorinated, chloroform is generally the most common by-product generated, while the majority of other DBPs are found in trace amounts, typically less than 1 μgl^−1^ (Nieuwenhuijsen et al., 2000). Production of DBPs that accompanies chlorine disinfection is an issue of great concern due to the increase in significant human health risks, such as cancer of the bladder and adverse associated reproductive outcomes (EPA, 2005; Richardson et al., 2007). This has made the development of alternative disinfectants a necessity due to the mutagenicity, carcinogenicity, and teratogenicity of the DBPs formed during chlorine disinfection in a bid to meet up with the regulation of DBPs (Gall et al., 2016).

### Monochloramination

Many chemical reactions occur from the interaction between chlorine and the aqueous solution of ammonia (Equations 3 and 4), which depends on the concentration, temperature, and pH of the aqueous solution. The mechanism of the formation of chloramine from the reaction between chlorine and aqueous ammonia is a function of the molar concentration of ammonia and chlorine (Equations 5 and 6; Kumar et al., 2020).


(3) $${\mathrm{Cl}}_{2}+H_{2}O↔\mathrm{HOCl}$$

(4) $$\mathrm{HOCl}+{\mathrm{NH}}_{3}\to {\mathrm{NH}}_{2}\mathrm{Cl}+H_{2}O$$

(5) $$\mathrm{HOCl}+{\mathrm{NH}}_{2}\mathrm{Cl}\to {\mathrm{NHCl}}_{2}+H_{2}O$$

(6) $$\mathrm{HOCl}+{\mathrm{NHCl}}_{2}\to {\mathrm{NCl}}_{3}+H_{2}O$$

Organic chloramine is the end-product of the reaction between chloramine and several amino acids. At a suitable temperature and pH, the organochlorine compound is formed at the interaction of chloramine and cell component which include alanine, tyrosine, and glycine. Monochloramine (NH_2_Cl) is a desirable disinfectant because; unlike free chlorine, it does not readily interact with natural organic matter to produce controlled DBPs [total trihalomethanes (TTHM) and haloacetic acids (HAA5); Lytle et al., 2021]. On the contrary, NH_2_Cl has been used by many drinking water systems in the United States as a secondary disinfectant to reduce the generation of DBPs and the growth of biofilms (Cromeans et al., 2010). The inactivation of bacteria by NH_2_Cl as well as their resistance to NH_2_Cl has been reported when extra and intracellular antibiotic resistance genes (eARG and iARG) were employed in both intracellular and extracellular components (He et al., 2019).

The inactivation rates of NH_2_Cl disinfection on AdV2, 40, and 41, E-1 and E-2, CVB3 and B5, and Murine Norovirus (MNV) have been compared by Cromeans et al. (2010). Disinfection with NH_2_Cl was most effective against E-1 and least effective against E-2 and AdV 2. However, to attain 4log inactivation of CVB5 and E-2, Ct values of 900 mg × min L^−1^ and 1,500 mg × min L^−1^ were required. At pH 7, the authors reported that NH_2_Cl was the most effective against AdV2, CVB5, and E-1. Unlike chlorine disinfection, NH_2_Cl disinfection showed more variation in virus inactivation rates (Cromeans et al., 2010). The outcome of the investigation revealed that NH_2_Cl efficacy data for different viruses should be integrated into the NH_2_Cl inactivation modeling and system design. In addition, no single disinfection method is effective in the inactivation of all types of viruses.

Kahler et al. (2011) examined the disinfection efficiency of NH_2_Cl on CVB5, E-11, MNV, and HAdV2 in untreated groundwater and two partially treated surface water. The authors reported that this method was most effective for MNV, followed by CVB5, while it was least effective for HAdV2 and E-11. The study indicated that the water quality impacts the inactivation of viruses; hence, this should be considered while developing monochloramination designs. According to Gall et al. (2016), at pH 9, Ct values of approximately 13,000 mg × min L^−1^ at 5°C and above 5,000 mg × min L^−1^ at 15°C are sufficient to attain 4log inactivation of HAdV. The examination of the different stages in the replication cycle of HAdV was conducted to understand the mechanism of inhibition of NH_2_Cl by Gall et al. (2016). Therefore, the authors concluded that there is a possibility of the inhibition of a replication cycle action after binding, although it was claimed that this would have occurred before the early viral protein synthesis.

Likewise, the effect of NH_2_Cl was examined in human norovirus (hNoV) GI, and GII, in secondary wastewater and phosphate buffer (PB). Using RT-qPCR as a method of detection, <0.5log_10_ reductions of all viruses at *C*t values were up to 450 mg × min L^−1^ except for hNoV GI, where 1log_10_ reductions at *C*t values of <50 mg × min L^−1^ for NH_2_Cl in wastewater were recorded. There was a comparable resistance to monochloramine by hNoVGI and MNV with 2log_10_ RT-qPCR reductions ranging from 300 to 360 mg × min L^−1^ in PB (Dunkin et al., 2017). The results revealed that there is an occurrence of genogroup dependent resistance pattern in hNoVs.

Because of poor disinfection properties and ineffectiveness against spore-forming waterborne pathogens like *Cryptosporidium*, chloramine is not utilized as a primary disinfectant (Kumar et al., 2020). When compared with chlorine, it is less virucidal (Dunkin et al., 2017), and a longer contact time is needed to attain similar disinfection efficiency because the active ingredient in chloramine, which is hypochlorite, is released slowly (Chen et al., 2021b). In other words, it is a less effective disinfectant than chlorine because it requires more exposure time to inactivate many waterborne pathogens, including enteric viruses.

### Chlorine dioxide

In recent years, there has been a significant increase in the usage of gaseous chlorine dioxide as a disinfectant for drinking water. Chlorine dioxide (ClO_2_) is typically utilized for water disinfection at 0.1 and 5.0 mgl^−1^ concentrations. It is a volatile gas created *in situ* by mechanical generators utilizing acid-based or electrolytic processes (Ma et al., 2017). It is employed as an oxidizing agent (Symons, 1981) to break down biofilm in pipes and tanks (Michael et al., 1981), and it can only react in water *via* oxidation with a low generation of THM. It oxidizes organic components derived primarily from oxidized by-products and a small quantity of chloro-organic molecules, whereas chlorine interacts with substances through oxidation and electrophilic substitution (WHO, 2002; Totaro et al., 2021). Furthermore, chlorine dioxide lowers the development of harmful halogenated disinfection by-products; however, at concentrations of 0.5 mgl^−1^, it also produces the organic halides chlorite/chlorate as well as tastes and odors (Thurston-Enriquez et al., 2005).

The disinfection impact of chlorine oxide on algae, protozoa, biofilms, bacteria, and viruses was also examined (Aieta and Berg, 1986). The inactivation process of viruses is not like other cells because the time of inactivation is shorter than that of bacteria even under similar conditions. This could be attributed to the less complex structure of viruses compared to bacteria. Chlorine oxide gas acts on the genome of the non-enveloped viruses, whereas in enveloped viruses, it interacts with one or more of the cysteine, tyrosine, and tryptophan amino acid residues of the spike proteins as it does not need to invade the enclosed viral surface (Noss et al., 1986; Noszticzius et al., 2013; Kály-Kullai et al., 2020). Factors that have significant effects on the inactivation rates of viruses using ClO_2_ include dosage, time, pH, and temperature (Berman and Hoff, 1984). When they are treated with 1.0 mg/l of chlorine oxide, enveloped viruses are inactivated readily compared to non-enveloped viruses (Sanekata et al., 2010). The adherence of the virus to the host cell is prevented *via* the disinfection action on the proteins of the viral envelope, and consequently, cell invasion and infection were prevented (Li et al., 2004). According to Noss et al. (1986), the premise of the disinfection process is a viral protein component; inactivating the coat viral protein results in the inhibition of the virus capacity to attack the host cells. Table 2 shows the inactivation efficiency of selected viruses by chlorine dioxide.

**Table 2:** Inactivation efficiency of selected viruses by chlorine dioxide.

Enteric virus	*C*t value	Inactivation efficiency	Conditions	Reference
Feline calicivirus	0.18 mg min L^−1^	4log	pH 8, 15°C	Thurston-Enriquez et al. (2005)
Feline calicivirus	9.59 min	4log	0.4 mg/l, pH 7.0, 20°C	Zoni et al. (2007)
Feline calicivirus	2 min	0.25log	1.0 mg L^−1^ ClO_2_	Sanekata et al. (2010)
Murine norovirus	0.25 mg min L^−1^	4log	pH 7.2, 5°C	Lim et al. (2010)
Coxsackievirus B5	2.41 min	4log	0.4 mg L^−1^ ClO_2_, pH 7.0, 20°C	Zoni et al. (2007)
Hepatitis A virus	19.58 min	4log	0.4 mg L^−1^ ClO_2_, pH 7.0, 20°C	Zoni et al. (2007)
Enterovirus 71	3.93 mg min L^−1^	4log	pH 7.2, 20°C	Jin et al. (2013)
Echovirus 11	1.0 mg min L^−1^	6log	pH 7.4	Zhong et al. (2017)
Human rotavirus	1.21 mg min L^−1^	4log	pH 7.2, 20°C	Xue et al. (2013)
Adenovirus type 40	0.12 mg min L^−1^	4log	pH 8, 15°C	Thurston-Enriquez et al. (2005)
Human Adenovirus	2 min	1.5log	1.0 mg L^−1^ ClO_2_	Sanekata et al. (2010)

Furthermore, Thurston-Enriquez et al. (2005), examined the inactivation efficiency of ClO_2_ within the range of 0.67–1.28 mgl^−1^ on AdV40 under pH values of 6 and 8 at 5°C and 15°C. The disinfection effectiveness of chlorine oxide improved at higher temperatures and pH values. Higher inactivation rates were reported at 15°C and pH 8 compared to other investigated conditions (Thurston-Enriquez et al., 2005). The effect of ClO_2_ at an initial concentration of 5 mgl^−1^ was elucidated on HAV, and the disinfectant could not eliminate the infectivity after 60 min; however, the virus was completely inactivated after 10 min having increased the concentration to 7.5 mgl^−1^. The 5′-non-translated region (5’NTR) of the virus’s genome was damaged by the disinfectant, which hindered the replication, and interaction with viral proteins and prevented adherence to the host’s cells (Li et al., 2004). A faster inactivation rate of 30 s at 0.8 mgl^−1^ and 5 min at 0.4 mgl^−1^, respectively, for HAV was reported by the Department of Public Health of Parma (Li et al., 2004; Zoni et al., 2007).

Feline Calicivirus (FCV) was inactivated completely by ClO_2_ in 30 min at 0.2 mgl^−1^ as reported by Alvarez and O’Brien (1982). In the same vein, at the same concentration and contact time, CVB5 was inactivated completely. Interestingly, at 0.2 mgl^−1^ of ClO_2_ and a reduced contact time of 4 min, complete inactivation of CVB5 was reported (Alvarez and O’Brien, 1982). Thurston-Enriquez et al. (2005) discovered that treating EV71 with ClO_2_ for more than 30 min (0.5 mgl^−1^), 25 min (1.5 mgl^−1^), and 15 min (2.0 mgl^−1^) resulted in complete inactivation of the virus. Similarly, at 4.92 mgl^−1^ of ClO_2_ for 1 min, higher rate of inactivation was reported at pH 8.2 compared to pH 5.6 while inactivation occurred rapidly at 36°C compared to 4°C or 20°C. The effectiveness of ClO_2_ for the inactivation of EV71 was dependent on both temperature and pH. Likewise, it has been found that the inactivation of AdV40 and FCV by ClO_2_ is more significant at 15°C than at 5°C (Thurston-Enriquez et al., 2005).

Chlorine dioxide is connected with DBPs such as chlorite and chlorate formation during water disinfection. As a disinfectant, ClO_2_ is commonly used for pre-oxidation followed by post-chlor(am)ination to reduce the production of chlorite and chlorate as neurotoxicity could result from a high dose of ClO_2_ (Chen et al., 2021b). The unstable nature of ClO_2_ prevents its storage; hence, it must be manufactured on-site and then added to water (Collivignarelli et al., 2018). Also, ClO_2_ is more biocidal when compared with chlorine and chloramines; however, it causes organoleptic abnormalities in treated water (Ngwenya et al., 2013), which makes this disinfectant less suitable for purification.

### Ozonation

For over a century, ozone (O_3_) has been used in drinking water treatment and chemical oxidation. It is used to substitute chlorine for disinfection in some parts of the world (Choudhury et al., 2018). Ozone is a bluish gas with a strong odor. It is an exceedingly reactive and unstable allotrope of oxygen (Rekhate and Srivastava, 2020). It is one of the most potent disinfectants available, and effective against practically all sorts of waterborne infections (Wolf et al., 2018). It is partially soluble in water and interacts with organic particles found in bacteria, viruses, and protozoa cells. The development of the organism’s cytoplasmic protein is inhibited as a result of the reaction between the ozone and the cell’s plasma membrane (Kumar et al., 2020).

Bacteria, viruses, protozoa, and prion protein as well as other pathogens such as *Cryptosporidium*, *Giardia* cysts, parvum oocysts, and *Legionella* that are resistant to chlorine are known to be effectively inactivated by ozone (Von Gunten, 2003; Betancourt and Rose, 2004; Passos et al., 2014; Li et al., 2017; Xi et al., 2017). More specifically, the effectiveness of ozone for the removal of viruses in water has been documented (Tondera et al., 2015; Wolf et al., 2018; Wang et al., 2018b), and this is usually through the oxidation of nucleic acids or promotion of protein coagulation in the viral particle (Tyrrell et al., 1995; Gomes et al., 2019b). Besides the inactivation of waterborne pathogens, O_3_ is employed to control taste and odor and the chemical oxidation of contaminants in drinking water (Chen et al., 2021b).

According to the Environmental Protection Agency (Agency, 1999), O_3_ at 0.1 mgl^−1^ outperforms chlorine at 2.0 mgl^−1^ due to faster reaction times in terms of disinfection with no regrowth of microorganisms. Ozone has been adopted recently in wastewater treatment with the primary goal of reducing micropollutants in secondary wastewater effluent and their impact on the aquatic environment (Wolf et al., 2018). Many potable reuse systems incorporate ozone as an essential component, for pre-oxidation of organic materials in the effluent, micropollutant reduction, and disinfection (Gerrity et al., 2013). In addition, due to its ability to reduce membrane fouling (Stanford et al., 2011), ozonation is commonly employed for membrane pre-treatment (Cheng et al., 2016).

In a bid to achieve improved disinfection using ozone, biologically activated carbon, free chlorine, or other catalysts have been incorporated with ozone. Gomes et al. (2019a), integrated volcanic rocks as catalysts into ozone, resulting in the complete removal of NoV GI, GII, and JC Polyomavirus (JCPyV). However, after 150 min, JCPyV remains inactivated. Also, an increase in the amount of ozone led to a surge in the disinfection of MS2 from 2.1 to 6.8log when ozone, coagulation, and ceramic membrane filtration were combined for the removal of viruses for water reclamation (Im et al., 2018).

Ozone can be used more effectively for viral inactivation in water than traditional water treatments such as mechanical treatment, aerated grit chamber, activated sludge, and reactors (Wang et al., 2018b). However, there could be a reduction in the inactivation with changes in some operational parameters, such as a lower temperature or an increase in pH. In clearer terms, ozone’s attenuation rate and oxidation ability are affected by a rise in pH and vice versa. In addition, other factors that could reduce the inactivation of viruses in water using ozone include the presence of particles, organic matter, and co-existing ions (Cai et al., 2014). Thus, the movement of ozone molecules is enhanced by an increase in temperature, resulting in the attachment of organic particles to the virus, which shields the viruses from ozone molecules and affects inactivation. In essence, the efficiency of ozone disinfection is reduced when a substantial quantity of ozone is consumed due to the presence of dissolved organic matters (Chen et al., 2021a).

The formation of bromate during disinfection from the oxidation of bromide ions in water (Chen et al., 2021a) a kind of DBPs is a major concern when significant ozone exposures are needed for pathogen inactivation (Gomes et al., 2019b). The instability and poor solubility of ozone in water is a setback for its application in practice; hence, its on-site production is required for its utilization (Rekhate and Srivastava, 2020). Therefore, there is a high operation cost associated with using ozone for disinfection in addition to maintenance costs linked to the use of ozonation equipment (Collivignarelli et al., 2018).

## Emerging methods

### Photocatalytic disinfection

The interaction between a photocatalyst (reaction initiator) and the aquatic medium (disinfection target) is known as photocatalytic disinfection; hence, both components of the photocatalyst and the properties of the aqueous medium are important factors in the reaction (Schneider et al., 2014; Smith and Rodrigues, 2015). The process of photocatalytic oxidation entails the production of electron–hole pairs by the irradiation of a semiconductor (such as TiO_2_) with appropriate light. As a result of the irradiation, electrons (e-) are stimulated into the conduction band (CB), leaving a hole (h+) in the valence band (VB). Thereafter, the duo of e−/h+, which are the charge carriers, migrates to the photocatalyst’s surface/interface and takes part in the redox processes. These charge carriers start a chain of events that result in the production of ROS. The ROS formed include singlet oxygen, superoxide radicals, hydrogen peroxide, hydroxyl radicals, and perhydroxyl radicals. These highly reactive chemicals are particularly important because they can engage in cellular component oxidation, microbial cell content release, and other activities (Cabiscol Català et al., 2000; Reddy et al., 2016).

Photocatalytic disinfection is very effective for the inactivation of bacterial species. TiO_2_ nanotubes were used for the inactivation of *E. coli* at the rate of 10^6^ CFU/ml under 10 min (Baram et al., 2009). Daels et al. (2015) also reported the effective inactivation of 76% of the overall bacterial colonies within 6 h of exposure time using TiO_2_-functionalized membranes. Also, Li et al. (2015) utilized Ag-TiO_2_ to disinfect *Pseudomonas aeruginosa.* Similarly, photocatalytic disinfection was also used to inactivate fungus and algae (Tatlıdil et al., 2011; Lee et al., 2015).

Over the last few years, nanostructured TiO_2_, the most notable studied photocatalyst, has been actively investigated for photocatalytic viral disinfection. Degussa P25 was the most preferred photocatalyst in all these investigations because of its strong photoactivity, long-term stability, relatively low toxicity, and inexpensive cost (Zhang et al., 2015; Wang et al., 2018a). Viral inactivation kinetics is affected by TiO_2_ crystalline structures which are either anatase or rutile. Sjogren and Sierka (1994) began the pioneering work on the inactivation of MS2 through photocatalytic viral disinfection by using TiO_2_ photocatalyst. Thereafter, TiO_2_ and TiO_2_ -based photocatalysts, metal-containing photocatalysts apart from TiO_2,_ and metal-free green photocatalysts have been employed to achieve improved antiviral disinfection effects (Table 3). Sato and Taya (2006) studied the impact of TiO_2_ particle crystalline structures on their virucidal ability. The authors achieved a maximum viral inactivation efficacy when both anatase and rutile TiO_2_ particles were combined with a 70% anatase ratio, having anatase and rutile phases that is 2 and 11 times more than TiO_2_, respectively_._

**Table 3:** Summary of parameters, inactivation efficiency, and photocatalysts used for viral disinfection in water.

Virus	Photocatalyst	Catalyst loading (mg L^−1^)	Virus level (PFU/ml)	Light source	Inactivation efficiency/time	References
Murine norovirus	TiO_2_	10	1 × 10^8^	UV lamp	3.3log/ 24 h	Lee et al. (2008)
MS-2 bacteriophage	TiO_2_	–	2 × 10^5^	4 W BLB lamp	2log/109 min	Cho et al. (2011)
Phage f2	TiO_2_	1,000	10^10^–10^11^	6 W black light Lamp	6log/15 min	Zuo et al. (2015)
Phage MS2	Mn-TiO_2_	100	1 × 10^5^	150 W Xe ozone-free lamp	4log/60 min	Venieri et al. (2015)
Phage MS2	g-C_3_N_4_	150	1 × 10^8^	300 W Xe lamp	8log/300 min	Li et al. (2016)
Phage Qβ	Rh-SrTiO_3_	3,000	5 × 10^7^	Vis	5log/120 min	Yamaguchi et al. (2017)
Phage MS2	FeO	5	1 × 10^6^	Simulated solar	5log/30 min	Giannakis et al. (2017a)
Phage f2	TiO_2_	100	>20	4 W UV-lamp	5-6log/160 min	Cheng et al. (2018b)
Bacteriophage f2	Cu - TiO_2_ Nanofibers	50	1 × 10^4^	Xe lamp	4.0log/120 min	Zheng et al. (2018)
Bacteriophage f2	Ag_3_PO4/g-C_3_N_4_	100	3 × 10^7^	8 W UVA lamp	6.5log/80 min	Cheng et al. (2018a)
Phage MS2	g-C_3_N_4_	135.4	1 × 10^8^	Xe lamp	8log/240 min	Zhang et al. (2018b)
MS2	g-C_3_N_4_/EP	0.06	1 × 10^8^	300 W Xe lamp	8log/240 min	Zhang et al. (2018a)
Bacteriophage f2	Cu - TiO_2_ Nanofibers	10	1 × 10^8^	Xe lamp	>5log/240 min	Cheng et al. (2019)
HAdV-2	O-g-C_3_N_4_/HTCC	3	–	7 W white LED Lamp	5log/120 min	Zhang et al. (2019b)
Norovirus	Cu- TiO_2_	–	2.89 ± 0.11 log 10	UVA-LED	5log/60 min	Moon et al. (2020)
HAdV-2	g-C_3_N_4_/H_2_O_2_	100	1 × 10^5^	300 W Xe lamp	2.6log/150 min	Zhang et al. (2021)
MNV	TiO_2_	300	1 × 10^6^	4 W Blacklight Blue Lamps	1.4 × 10^−5^/32 min	Kim et al. (2021)

To achieve increased viral inactivation in drinking water, researchers have embarked on enhancing both metal and metal oxides. TiO_2_ photocatalysis was improved by loading the photocatalyst with nano-sized silver (nAg) by Liga et al. (2011), as a result of the several benefits attached, which include suppressed charge recombination in TiO_2_ by trapping the excited electrons, increased surface area for virus adsorption, and Ag^+^ release for viral inactivation. The combination of TiO_2_ and nAg facilitated increased viral disinfection efficiency, as 6.2-log MS2 was inactivated within 2 min of UV irradiation using nAg/TiO_2_ and was > 6 times quicker compared to the unmodified TiO_2_.

Furthermore, a variety of effective, metal-containing, visible-light-active photocatalysts, as well as TiO_2_-based photocatalysts, for instance, plasmon-induced viral disinfection by Ag-AgI/Al_2_O_3_ (Hu et al., 2010) and platinum-tungsten oxide (Pt-WO_3_; Takehara et al., 2010), have been developed to enable solar-driven inactivation of waterborne viruses. Bacteriophage MS2 titer of 2 × 10^6^ PFU/mL was reduced to <5 PFU/mL under visible-light radiation for 3 h when graphene oxide (GO) sheets were integrated into WO_3_ films to give graphene-WO_3_(G-WO_3_) films (Akhavan et al., 2012). Wustite, Maghemite, and nano-Maghemite which are iron oxides were explored for their photocatalytic antiviral activity using solar irradiation. Wustite demonstrated the highest disinfection efficiency with a record of 5log MS2 under 30 min, while 2.6log MS2 inactivation was achieved within 120 min (Giannakis et al., 2017a). Some of the distinct advantages of using iron ore minerals to inactivate microbial contaminants include low-cost material, availability, earth abundance, visible-light reaction owing to the presence of iron components, and simple magnetic recovery from water. Therefore, natural photocatalysts based on iron have a potential for industrial manufacture and real-time utilization for waterborne viruses’ disinfection (Zhang et al., 2019a).

Moreover, fullerene-based photocatalysts have been developed to improve the disinfection and inactivation of waterborne viruses. Novel C_60_ derivatives with diverse functional groups such as eNH_3_^+^, –COOH, or –OH-terminals were developed, with improved water stability. There is a possibility that the electrostatic bond between positively charged C_60_ derivatives and negatively charged MS2 viruses was responsible for the outstanding viral inactivation activity demonstrated by the cationic aminoC_60_ when compared to the commercial TiO_2_ P25 (Lee et al., 2009; Cho et al., 2010; Lee et al., 2010; Moor and Kim, 2014; Moor et al., 2015). In addition, aminoC_60_ was found to be reactive when exposed to visible light from fluorescent lamps as well as sunlight (Cho et al., 2010). Furthermore, an organic linker with an amide group was utilized to link the C_60_ derivatives onto functionalized SiO_2_ surface all in a bid to reduce their accidental discharge to water (Lee et al., 2010), leading to a significant improvement of the viral inactivation.

To develop an outstanding photocatalyst with a very high viral disinfection efficiency, Hu et al. (2012) introduced aptamers on the edge of GO and the composite novel antiviral material through energy transform/electron transfer efficiently inactivated viruses as it damaged nucleic acids and viral proteins using visible-light radiation. Aptamers that were integrated into GO increased the GO nanosheets’ stability and bind to targets precisely. Graphitic carbon nitride (g-C_3_N_4_) became an attractive candidate for photocatalytic viral inactivation due to its biocompatibility with insignificant toxicity, resistance to photo-corrosion, air oxidation at increased temperature, and chemical stability in solvents such as acids and bases (Cao et al., 2015; Zheng et al., 2017).

Matsuura et al. (2021) examined the ability of light-emitting diode (LED)-activated TiO_2_ fixed on a glass sheet to inactivate SARS-CoV-2 in aerosol and liquid. They reported that SARS-CoV-2 was inactivated with a reduction in its infectivity by 99% after 20 min and 120 min of interaction in aerosol and liquid, respectively. Hence, it was deduced that the photocatalytic interaction mediated by TiO_2_ with the SARS-CoV-2 virus was dependent on time. The impacts of TiO_2_ photocatalyst on SARS-CoV-2 virion include reduced virion count, increased virion size, decreased particle surface spike structure, and destruction of viral proteins and genome. The authors believed that the degradation of the spike protein of SARS-CoV-2 by the photocatalytic reaction of TiO_2_ could inactivate the virus irrespective of the mutation in the protein.

However, one of the major limitations of using TiO_2_ in water disinfection is the reaction kinetics; therefore, more studies should focus on improving the TiO_2_ inactivation efficiency and the rate of solar energy utilization toward viral inactivation. Furthermore, magnetically separable materials that facilitate the removal of used photocatalysts from aquatic environments are another obstacle for the semiconductor-based photocatalytic viral disinfection method. Thus, floating photocatalysts are interesting candidates for enhancing the use of light/oxygen to generate more ROS (Habibi-Yangjeh et al., 2020).

Another significant drawback of the photocatalytic disinfection method for the inactivation of waterborne viruses is the high level of charge recombination in TiO_2_ and low photocatalytic activity. In addition, the broad bandgap of TiO_2_ (3.2 eV for anatase and 3.0 eV for rutile) enables the material to operate solely on UV light accounting for approximately 4% of solar energy used for photocatalytic disinfection. The majority of photocatalysts that have been shown to have outstanding antiviral action contain (heavy) metals, and the accidental release of hazardous metals (e.g., Cu, Ag) into treated water presents serious health issues (Zhang et al., 2019a).

### Cavitation

Cavitation is the production of tiny vapor bubbles (cavities) within a liquid that was originally uniform and an abrupt drop in pressure causes this quick physical process. Cavities are formed due to the disruption of the liquid medium at one or more sites and their shapes are majorly influenced by the flow structure. The unstable vapor structures frequently collapse suddenly as they approach a section of elevated pressure. Strong shear flows, jets, high local temperatures, shock waves, rapid depressurization, and supersonic flow are all possible outcomes of the collapse (Shamsborhan et al., 2010; Kosel et al., 2017). In general, there are two types of cavitation: hydrodynamic cavitation (HC) and acoustic cavitation (AC). The process which results in a drop in the local pressure differentiates the two; however, the mechanisms that regulate the hydrodynamic bubble and the acoustic bubble are essentially similar. In AC, the propagation of acoustic waves achieves the required low pressures to cause the disruption of the liquid and lead to cavitation. In contrast, the liquid’s current speed in HC will generate a reduced local pressure below saturation point for a liquid temperature, resulting in cavitation formation (Zupanc et al., 2019).

The precise mechanism by which cavitation inactivates viruses is not fully comprehended yet as few studies have evaluated the impact of cavitation on waterborne viruses (Filipić et al., 2022). The inactivation of viruses was known to be caused by heat and high pressure, and OH^−^ was linked to damage of viral capsid proteins (Chen et al., 2021b). According to Su et al. (2010), virus inactivation could be due to the damage caused during cavitation either to the exterior protein capsid or the recognition sites situated on the exterior of the capsid. Kosel et al. (2017), opined that the alteration of the viral capsid or genome by OH^−^ produced during cavitation combined with mechanical impacts could be attributed to virus inactivation.

Dular et al. (2016), assessed the effect of HC on the decrease of rotavirus (RV), in a Venturi cavitation chamber using a pulsating system. The treatment reduced the RV concentration by 75% using RT-qPCR. In a similar Venturi constriction, the effect of HC on MS2 infectivity was investigated by Kosel et al. (2017), and it was reported that there was a 4.8log reduction per litre. The authors also mentioned that the viral inactivation could be linked to the damage of the host’s recognition receptors situated on the virus surface due to the OH^−^ radicals generated from cavitation. Also, they posited that OH^−^ radicals were formed because cavitation could be responsible for the observed damage. Furthermore, they suggested that high shear forces inside the cavity could be another factor responsible for additional damage to the virus. Filipić et al. (2022), recently evaluated the inactivation of potato virus Y (PVY) in a liter of water using HC where inactivation was achieved after about 125 to 500 HC passes. It was observed that there was rapid and severe damage to the protein capsid of the virus compared to the genomic RNA, and this could be a major contributory factor to the virus inactivation. The authors further observed that strong oxidants such as O_2_, OH^−^, and H_2_O_2_ were not really involved in the virus inactivation; hence, this suggested that mechanical effects are possibly responsible for the inactivation.

Although hydrodynamic cavitation is a less expensive form of disinfection than AC, it costs more than chlorination and ozonation (Holkar et al., 2019). Also, it requires a continuous energy supply, and there is a limit to how much water can be treated using this process (Dular et al., 2016). Due to significant operational expenses, HC disinfection technology is currently in the laboratory stage (Pichel et al., 2019), and is yet to be implemented in large-scale applications (Sun et al., 2020).

### Electrochemical disinfection

The primary basis of the electrochemical disinfection method is the oxidation ability of disinfectants in the electrode layer or the bulk of the electrode (Bruguera-Casamada et al., 2016; Ghernaout, 2017). This disinfection method is characterized by the production of intermediates, and it is grouped into two categories: direct anode oxidization, and indirect product oxidation. The transfer of electrons between the electrode and the target material without using poisonous chemicals and other organic molecules is the basis of inactivation when direct anode oxidation is performed. In contrast, concentrated saline solution is needed for anode oxidation, and therefore the accumulated molecules and ions (e.g., H_2_O and Cl) at the electrode are oxidized for the formation of chlorine-active substances (for instance, Cl_2_, HOCl, and ClO_3_) as well as oxygen-active substances (e.g., oscillation of [O(3P)], H_2_O_2_, O_3_). To oxidize and remove the target substance, intermediate products are critical in transporting the electrons from the target material to the surface of the electrode (Panizza and Cerisola, 2005; Santana et al., 2005; Yang et al., 2018; Jung et al., 2020). The main cause of the inactivation of microorganisms when electrochemical disinfection is employed is damage to the intracellular enzyme system of the microbes (Long et al., 2015).

Boron-doped diamond electrodes have also been employed as a disinfectant in a sequential electrocoagulation-electro-oxidation treatment system for the removal of viruses in water. Bacteriophages MS2, ΦX174, and human echovirus were reduced from the positive simulation effect in the physical decrease of coagulation-filtration, ferrous iron-based disinfection, and electro-oxidation disinfection (Heffron et al., 2019). Tu et al. (2021) reported the inactivation of SARS-CoV-2 virus in Na_2_CO_3_ aqueous solution using nickel foam as both cathode and anode. Inactivation rates of 95, 99, and 99.99% at 5 V were recorded within just 30s, 2 min, and 5 min, respectively. The inactivation rates were attributed to oxidation and degradation of the receptor binding domain (RBD) of the SARS-CoV-2 spike glycoprotein by the NiOOH anode surface formed *in situ* during the electrolysis.

Even though viruses are smaller and less complex physically than bacteria, they resist electrochemical treatment more strongly, limiting the method’s utilization, thereby necessitating further research (Drees et al., 2003; Chen et al., 2021b). Much attention has not been given to DBPs formation from electrochemical disinfection; however, some researchers now explore the possibility of by-products generation from electrochemical disinfectants and their possible toxicity levels (Jasper et al., 2017; García-Espinoza and Nacheva, 2019). Disinfection by-products that are produced during chemical disinfection processes might also be produced during electrochemical disinfection treatment depending on the electrode material used and applied voltage (Ghernaout, 2017).

## Comparison of disinfection methods

Common disinfection methods involve membrane filters, ultraviolet irradiation, chlorine, monochloramine, chlorine dioxide, ozone, and emerging disinfection treatments such as photocatalytic and electrochemical disinfection. The mechanism of disinfection for virus mitigation and inactivation is based on the contact between the viruses and disinfectants to break down the virus’s capsid protein and nucleic acid. This makes it impossible for viruses to spread to host cells and reproduce, while membrane filtration employs the size exclusion principle. The kind and primary concentration of the disinfectant and virus, as well as the pH, temperature, and treated water matrix (particles, dissolved oxygen, coexisting ions, and dissolved organic matter), remain the major factors militating against water disinfection.

Each disinfection method reviewed reduces the viral load before the effluent is discharged into the environment. However, the rate of viral inactivation differs, for instance, ozonation and UV irradiation are more effective for viral inactivation when compared with chlorination. Interestingly, UV irradiation is considered a clean disinfection technology due to its viral inactivation efficiency without forming DBPs. Furthermore, studies have revealed that RVs (Betancourt and Gerba, 2016), JC PyV, and echoviruses are better inactivated using chlorine compared to other waterborne viruses (Chen et al., 2021a). SARS-CoV-2 is inactivated by both chlorine and chlorine oxide (Brown et al., 2021; Hatanaka et al., 2021), as well as the emerging methods: photocatalytic disinfection (Khaiboullina et al., 2020), and electrochemical disinfection (Tu et al., 2021). However, the efficiency of cavitation for the disinfection of SARS-CoV-2 has not been investigated. Adenoviruses which have been reported to be resistant to UV irradiation (Augsburger et al., 2021), and monochloramination (Gall et al., 2016), are inactivated by both ozonation and photocatalysis disinfection (Chen et al., 2021a). Furthermore, Chen et al. (2021b) mentioned that inactivation of *E. coli*, *Clostridium perfringens*, *Vibrio cholerae*, and MS2 was faster using chlorine-active substances produced on-site compared to chlorine. Table 4 further summarized the virus removal/inactivation range, merits, and limitations of various disinfection methods discussed in this article. The log reduction value (LRV) shows the relative number of inactivated pathogens during the disinfection process (Equation 7).


(7) $$\log\mathrm{reduction}\;\mathrm{values}\left(\mathrm{LRV}\right)=\log_{10}\left(A/B\right)$$

where A is the number of viable pathogens before treatment while B is the number of pathogens after treatment (Mariita and Randive, 2021).

**Table 4:** Virus removal/inactivation range and the merits and limitations of the various disinfection method (adapted from Chen et al., 2021a).

Method	Removal/inactivation log	Merits	Limitations
Membrane filtration	0.5–5.9	Low energy cost, the potential for mobile treatment unit, does not require chemicals	Removal efficiency is unstable, a potential health risk for humans
Ultraviolet irradiation	0.09–5	No DBPs formation, less susceptible to pH and temperature, non-corrosive, ease of installation and operation, short contact time	Relatively high energy consumption, inefficient in turbid water
Chlorination	1- > 5	Simple to handle, cost-effective, residual in distribution	DBPs production, residual toxicity
Monochloramination	0.5–4	Stable residual, less odor, and taste issues	Weak disinfectant, less virucidal, long contact time
Chlorine dioxide	0.25–6	More effective than chlorine at higher pH, lowers DBPs formation	DBPs formation, organoleptic abnormalities
Ozonation	0.6–7.7	Effective disinfectant, short contact time, possible combination with various catalysts	DBPs formation, high operation and maintenance cost, non-stable and poor solubility, effectiveness is affected by water turbidity
Photocatalytic disinfection	1–8	Low cost of operation, possible reuse of catalysts, favorable catalytic performance	Accidental leaching of hazardous metals into treated water
Cavitation	<4	No DBPs formation, possible for incorporation into a continuous flow process	Energy-intensive and high operating cost, still at the developmental stage
Electrochemical disinfection	3.4–5	Easy to control, environment friendly	Possibility of DBPs formation, low selectivity, the high operating cost associated with electricity consumption

## Conclusion and perspectives

Disinfection is critical in the elimination of waterborne microorganisms for the discharge of safe water to the environment. However, viruses differ from other pathogens in that they react differently in treatment processes, resulting in differences in their fate and behavior in water. Therefore, this has led to the adoption of different disinfection techniques, all in a bid to achieve safe water that is void of viruses. Unfortunately, secondary pollution caused by the formation of DBPs associated with chemical disinfectants cannot be overemphasized; hence, it should be avoided during viral disinfection. Interestingly, single techniques of disinfection can be sequentially merged into one and utilized as one method.

Emerging disinfection technologies have the potential to significantly increase virus inactivation in water by utilizing synergistic effects of different disinfection methods to solve the problem of the persistence of waterborne viruses. Nevertheless, they are not void of toxicity issues and accompanying high cost of operation. For example, to reduce DBPs formation yet increase viral inactivation, UV irradiation could precede chlorination, and this would reduce the quantity of chlorine that is required. On the other hand, there could be formation of more DBPs if already chlorinated water is exposed to UV radiation. Furthermore, the feasibility of the application of these merged technologies to large-scale water treatment plants still hinders the adoption of these techniques on an industrial scale despite their undeniable benefits. Therefore, these techniques should be investigated on a pilot scale to ascertain their feasibility. Considering the foregoing, it could be logically proposed that natural biomaterials such as medicinal plants that are biocompatible, biodegradable, abundant, readily available, and cost-efficient with intrinsic health and safety benefits coupled with their significant antimicrobial attributes should be explored as potential novel disinfectants, and adapted for the inactivation of waterborne viruses.

## Author contributions

AL, AE, and FS conceived the review idea. AL wrote the first draft. AL, AE, and SS reviewed and edited the manuscript. All authors approved the final manuscript.

## Funding

This work was funded by the Water Research Commission (WRC) of South Africa (project no. K5/C2020-2021-00181). We were also supported by our institution, the Durban University of Technology, South Africa.

## Conflict of interest

The authors declare that they have no known competing financial interests or personal relationships that could have appeared to influence the work reported in this paper.

## Publisher’s note

All claims expressed in this article are solely those of the authors and do not necessarily represent those of their affiliated organizations, or those of the publisher, the editors and the reviewers. Any product that may be evaluated in this article, or claim that may be made by its manufacturer, is not guaranteed or endorsed by the publisher.
